# Microfluidic enrichment of proteolytic microbial consortia from sewage sludge

**DOI:** 10.1128/aem.00293-26

**Published:** 2026-07-10

**Authors:** Luca Potenza, Valentina Smacchia, Łukasz Drewniak, Tomasz S. Kaminski

**Affiliations:** 1 Laboratory of Microfluidics and Single Cell Analysis, Institute of Biochemistry, Faculty of Biology, University of Warsaw 49605, Warsaw, Poland; 2 Institute of Evolutionary Biology, Faculty of Biology, University of Warsaw 49605, Warsaw, Poland; 3 Institute of Bioengineering, Faculty of Biology, University of Warsaw 49605, Warsaw, Poland; Norwegian University of Life Sciences, Ås, Norway

**Keywords:** sewage sludge, microbial consortia, proteolytic activity, high-throughput screening (HTS)

## Abstract

Proteolytic microbial consortia are key drivers of protein hydrolysis in complex organic substrates. In anaerobic digestion systems, such as those used for biogas production from sewage sludge, this process constitutes the initial and rate-limiting step. Despite their importance, proteolytic microorganisms remain poorly characterized due to the complexity of environmental microbiomes and the limitations of conventional cultivation and screening methods. Here, we present a label-free microfluidic protocol for the high-throughput cultivation and characterization of proteolytic microorganisms. Single microbial cells are encapsulated in gelatin droplets and grown clonally, where proteolytic activity is detected through image-based analysis of droplet shape changes. Enrichment of individual proteolytic cultures is achieved using a separate microfluidic device that enables passive droplet sorting. Taxonomic characterization of sorted droplets by 16S rRNA gene sequencing revealed a fivefold higher number of amplicon sequence variants (ASVs), and a more diverse array of proteolytic strains were recovered compared with conventional skim milk agar screening (SMA). Taken together, this microfluidic workflow allows accurate and fast enrichment of proteolytic strains. Our approach advances the understanding of proteolytic communities in sewage sludge and opens new opportunities for targeted microbial recovery in waste-to-energy applications.

**IMPORTANCE:**

Proteolytic microorganisms drive the initial and rate-limiting step of protein degradation in anaerobic digestion systems, such as sewage sludge biogas production, yet their diversity and function remain poorly characterized due to the limitations of conventional cultivation methods. We present a label-free droplet microfluidic workflow that enables high-throughput, single-cell cultivation, functional screening, and selective enrichment of proteolytic microbes directly from complex communities. This approach substantially improves the recovery and diversity of proteolytic strains compared with traditional assays, providing a powerful tool to study hydrolytic consortia and to enhance microbial discovery for waste-to-energy and other biotechnological applications.

## INTRODUCTION

Biogas production represents a substantial component of current sustainable energy strategies, converting organic waste into methane-rich fuel and mitigating environmental pollution (1). Sewage sludge, a ubiquitous by-product of municipal and industrial wastewater treatment, is particularly attractive as an anaerobic digestion substrate due to its abundance and high organic content (2). However, the efficiency and stability of anaerobic digestion are frequently constrained by the hydrolysis step, the initial and rate-limiting phase during which complex polymers, such as proteins, polysaccharides, and lipids are broken down into soluble monomers (3, 4).

Recent research in biogas production has either highlighted the synergistic role of diverse hydrolytic microbial consortia in sludge hydrolysis (5, 6) or focused primarily on the degradation of lignocellulosic biomass (3, 7, 8). Nonetheless, protein degradation remains relatively underexplored, and the enrichment of proteolytic strains remains challenging; only a limited number of recent studies have begun to examine their diversity, ecological roles, and potential contributions to improving biogas production (9). In this context, proteolytic consortia represent a critical yet poorly characterized component of sludge hydrolysis. Although hydrolysis is widely recognized as the rate-limiting step in anaerobic digestion, protein hydrolysis constitutes a distinct bottleneck in sewage sludge, as proteins are degraded later and less completely than carbohydrates, thereby constraining methane yield (10). Accordingly, targeted enhancement of proteolytic activity has been shown to increase methane production (11).

Culture-independent approaches like 16S rRNA sequencing and metagenomics have broadened our understanding of sludge microbiome diversity. Yet, they provide only indirect evidence of enzymatic activity and fail to bridge the genotype-phenotype gap, particularly at the single-cell level, hindering our ability to link specific taxa to proteolytic functions in complex microbial samples (12). Conventional methods for studying proteolytic communities, such as plating on selective media or cultivation in liquid media followed by spectrophotometric assays using chromogenic or fluorogenic substrates, are inherently biased toward fast-growing or easily cultivable microbes. Moreover, artificial substrates may not accurately reflect *in situ* activity, limiting ecological relevance and functional discovery (13). The SMA assay is a common method for screening microbial protease activity. Protease-producing microorganisms hydrolyze casein, forming clear halos around colonies, while non-proteolytic strains leave the medium opaque. Halo size relative to colony growth provides a semi-quantitative proteolytic index. SMA is inexpensive, simple, and allows rapid visual discrimination of protease-positive strains in both pure cultures and mixed communities. However, halo formation depends on growth rate, colony morphology, and medium diffusion, potentially biasing results, and it only offers qualitative or semi-quantitative information without capturing full protease specificity or environmental activity. In addition, non-proteolytic mechanisms such as acidification can occasionally alter medium opacity, leading to false positives (14). Another popular method is represented by gelatin-based assays that detect protease activity as protease-secreting microbes hydrolyze gelatin, breaking down the gel and causing medium liquefaction. Gelatin serves as both a nutrient and a gelling substrate, and liquefaction provides a simple qualitative measure of proteolytic activity.

Droplet microfluidics offers a powerful alternative to traditional cultivation by encapsulating individual microbial cells in millions of picoliter-scale droplets that serve as isolated microreactors for growth and high-throughput activity-based assays. Non-targeted droplet cultivation of complex microbiomes has enabled recovery of greater microbial diversity than bulk culture, including rare and slow-growing taxa, but lacks the ability to selectively enrich specific functional traits (15–17). In contrast, only a few studies have reported targeted droplet microfluidic enrichment and characterization from environmental samples (18), and to date, no label-free droplet-based targeted enrichment has been described for proteolytic microorganisms. The strengths of droplet-based microfluidics comprise single-cell encapsulation, precise manipulation, analysis, and sorting, using several different detection modalities, among the most common: absorbance and fluorescence (19, 20). We previously introduced passive and image-based droplet microfluidic platforms for screening proteolytic microorganisms (21, 22), based on the encapsulation of single cells in gelatin droplets and sorting by droplet deformability at higher throughput than earlier deformability-based approaches (23). These advances highlight the potential of droplet microfluidics to link microbial function to single-cell phenotype and accelerate the discovery of proteolytic species relevant to biogas optimization. In this context, proteolytic consortia remain a key yet insufficiently characterized component of sludge hydrolysis, and this study contributes to ongoing efforts to better understand their composition and role in substrate degradation. Here, we present a droplet microfluidic workflow that integrates two previously developed label-free protocols exploiting changes in the mechanical properties of gelatin droplets to detect and isolate proteolytic microorganisms. This approach enables the enumeration and isolation of proteolytic bacterial cultures originating from single cells encapsulated in picoliter droplets. Following incubation, droplets were first analyzed using the induced droplet ovalization (IDO) method, in which image-based quantification of droplet deformation correlates with microbial proteolytic activity (21). Subsequently, droplets were sorted at high throughput using a deformability-based passive droplet sorter (DPDS) (22), enabling the enrichment and downstream taxonomic characterization of proteolytic microcultures. The performance of the proposed droplet-based method was compared with the SMA protocol to evaluate their respective effectiveness in isolating proteolytic consortia from sludge samples. The objectives of this study were to (i) apply high-throughput strategies for functional screening of proteolytic bacteria from complex environmental samples, such as sewage sludge; (ii) quantify, enrich, and identify proteolytic strains via 16S sequencing; and (iii) evaluate droplet-based protocols as scalable, label-free alternatives to conventional approaches, such as SMA screening.

## RESULTS AND DISCUSSION

**Fig 1:**
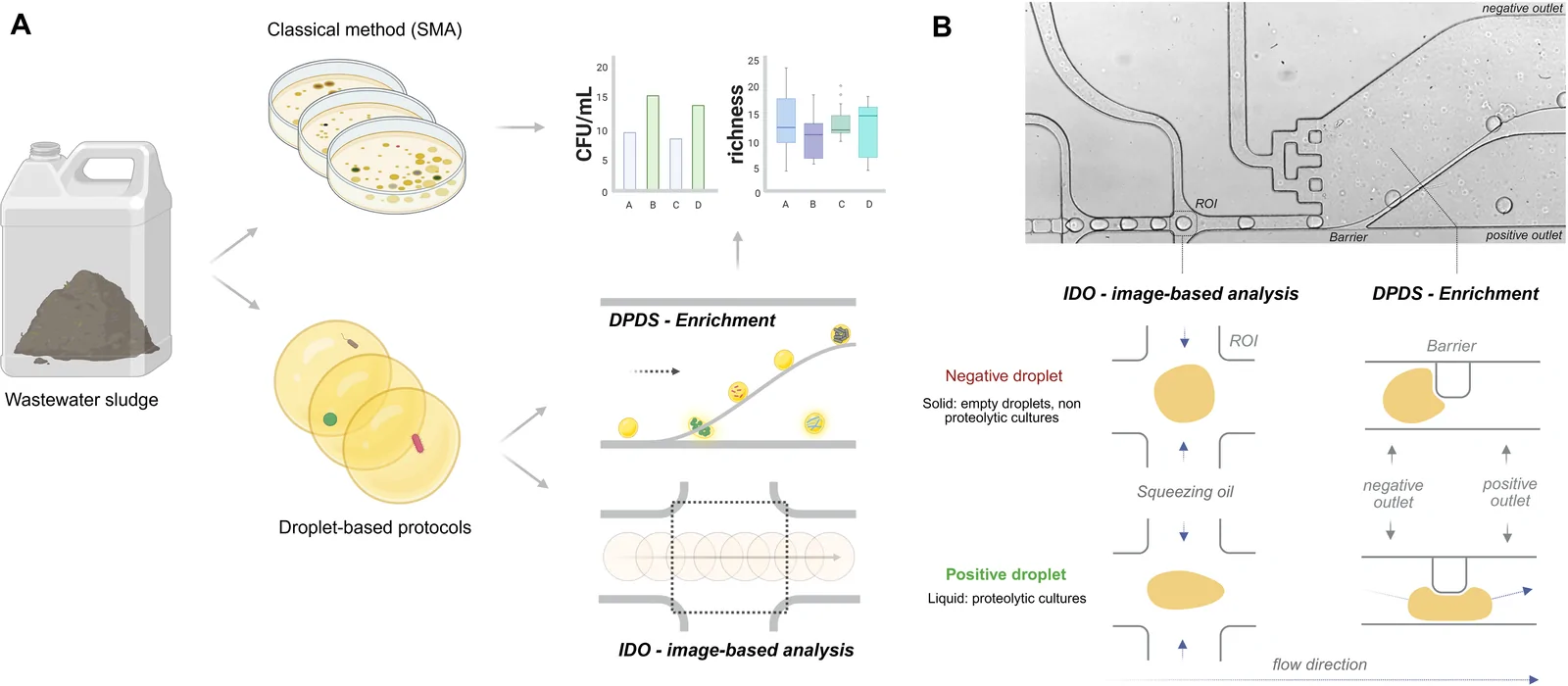
Environmental screening of microbial proteolytic activity. (**A**) Classical screening of sewage sludge was performed by plating microorganisms on skim milk agar (SMA), followed by manual quantification and analysis of colonies displaying proteolytic activity. In parallel, droplet-based microfluidic protocols enabled the encapsulation of single microbial cells into picoliter-sized compartments, where they were cultured and assayed for enzymatic activity in a high-throughput and automated manner. (**B**) In this workflow, gelatin droplets were first characterized using image-based induced droplet ovalization (IDO) analysis to quantify the abundance of proteolytic strains and were then passively sorted via the deformability-based passive droplet sorter (DPDS) devicefor the enrichment of proteolytic strains. This microfluidic protocol offered an effective means to assess microbial activity and diversity in environmental samples, while also serving as a direct benchmark against classical approaches.

We cultivated, enriched, and characterized proteolytic strains from sewage sludge, as outlined in Fig. 1A, and evaluated our novel droplet-based microfluidic workflow against a conventional protocol. Sludge was first resuspended and streaked on solid medium, while in parallel, single cells were encapsulated in gelatin droplets for clonal cultivation. In the traditional SMA assay, community analysis and colony isolation were performed manually, whereas the droplet-based system enabled automated, high-throughput processing. This setup allowed a direct comparison of outcomes between bulk and droplet-based methods.

We employed two complementary droplet microfluidic protocols to detect proteolytic activity at the single-cell level, Fig. 1B. In the IDO analysis, individual cells are encapsulated in gelatin droplets, and microbial protease production alters droplet viscoelastic properties, making the droplets more deformable in a flow-focusing device; these deformations, monitored by automated image analysis, correlate with the proteolytic activity of the encapsulated clonal cultures. Detection of droplet deformability enables label-free, high-throughput identification and enumeration of proteolytic strains (21). In the DPDS method, gelatin degradation by proteases triggers a solid-to-liquid phase transition, allowing passive microfluidic sorting of liquid droplets (proteolytic strains) from stiffer droplets (empty or non-proteolytic cultures) by flowing droplets over a microbarrier, where only liquid droplets are positively sorted without labels, optoelectronic components, or complex hardware (22). Finally, droplet samples were sequenced to assess taxonomic richness of sewage sludge. Together, these methods enable rapid, high-throughput analysis and enrichment of proteolytic microbial consortia from environmental samples. Although the screened droplets flowed through the same DPDS device, the image analysis method was specifically developed for large-scale automatic detection and quantification of proteolytic strains. This approach enabled accurate assessment of the proteolytic microcultures present within the emulsion at the second spacing junction of the device. The sorting step enabled the selective enrichment of proteolytic strains and was performed using the passive microbarrier structure positioned immediately downstream of the second spacing junction. Importantly, the image-based characterization and passive sorting experiments were conducted as two separate workflows using droplets derived from the same emulsion population. In the first experiment, image analysis was used for large-scale characterization and quantification of proteolytic microcultures, whereas in the second experiment the DPDS device was used for passive enrichment of proteolytic droplets. Consequently, the deformed droplets identified during image analysis were not subsequently isolated through the barrier structure, which explains the discrepancy in droplet numbers observed between the two stages of the proposed method. Although integration of image-based detection with active droplet sorting represents an interesting future direction for the IDO platform, such implementation would require the development of an image-based active droplet sorting system and therefore remains beyond the scope of the present study.

### Analysis of the abundance of proteolytic bacteria in sewage sludge using the IDO system

For this study, sewage sludge was selected as the environmental sample for microbial isolation. The sludge sample analyzed in this study was collected in summer (August 2024) and represents a different sample from that used in the previously described sewage sludge analysis conducted for validation of the IDO protocol. The validation study was performed using a winter sample (February 2025) collected from the same biogas facility. In our previous work, the sludge sample was already used primarily to validate a droplet-deformability, image-based method for analyzing proteolytic activity (21). In the present study, we analyzed a different wastewater sample to expand the enrichment protocol (22) by enabling automated and accurate enumeration of positive cultures, followed by their genomic identification, thereby broadening the applicability of a user-friendly droplet-based approach for screening microbial consortia.

**Fig 2:**
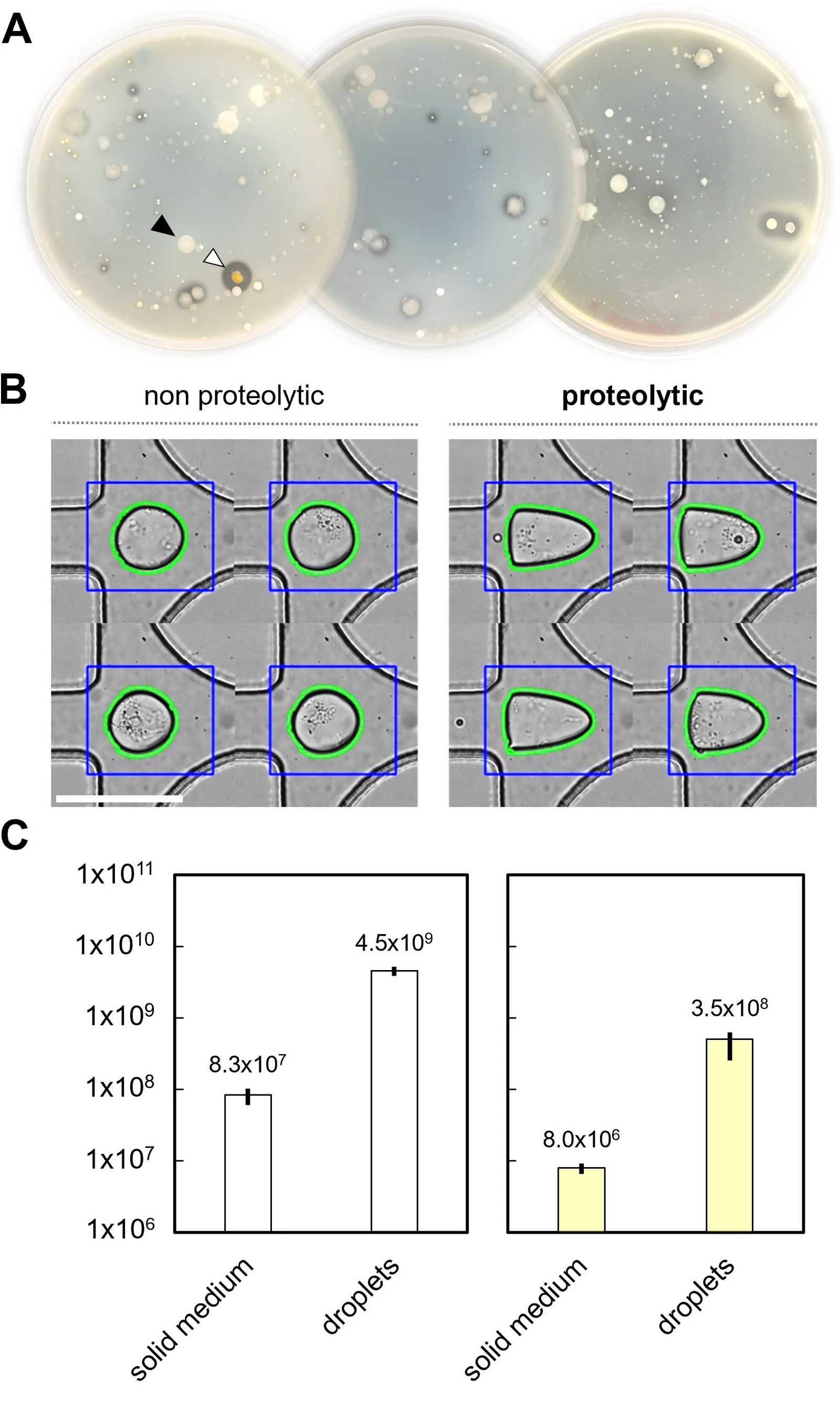
Comparative screening of microbial consortia using solid medium and droplet-based image analysis. (**A**) Representative SMA plates after 96 h of incubation, showcased phenotypic differentiation of microbial colonies. Colonies surrounded by transparent halos (an example is indicated by the white arrowhead) exhibit proteolytic activity visible due to gelatin hydrolysis, whereas colonies without halos (an example is indicated by the black arrowhead) correspond to non-proteolytic strains. (**B**) In the image-based analysis (IDO), proteolytic strains exhibit a distinct elongated droplet morphology, resulting from gelatin degradation and the consequent deformation at the DPDS focusing junction. In contrast, droplets carrying non-proteolytic strains retain a spherical morphology. Representative examples of droplets are shown, scale bar: 100 µm. (**C**) The bar plots illustrate how cultivation in gelatin droplets (λ ~ 0.2) resulted in approximately two orders of magnitude higher growth for both non-proteolytic (white bars) and proteolytic (yellow bars) strains compared with conventional SMA plating. This highlights the enhanced sensitivity of droplet-based microfluidic analysis relative to traditional solid-medium screening. Quantification of proteolytic and non-proteolytic strains was performed via SMA across serial dilutions from the same sludge suspension used for droplet generation; six replicates from the 10⁻³ dilution were enumerated. Droplet-based video analysis was performed by visually classifying 5,666 droplets from the same recordings used for automated IDO analysis.

As shown in Fig. 2A, SMA dishes display proteolytic colonies characterized by a transparent halo, whereas colonies lacking the halo were the non-proteolytic strains. Image analysis (IDO) identified positive droplets, which appeared oval-shaped when compressed in the second focusing junction of the sorter device, the DPDS, as shown in Fig. 2B. In contrast, non-proteolytic and empty droplets remained spherical due to the presence of gelatin within the droplets. The results from the different screenings revealed a substantially higher number of CFU/mL of proteolytic strains detected automatically through IDO analysis compared with the SMA protocol, Fig. 2C. Interestingly, single-cell encapsulation in droplets also yielded a greater number of non-proteolytic strains, despite the system not being specifically designed for this purpose. Interestingly, single-cell encapsulation in droplets primarily enhances the overall cultivable diversity, enabling the recovery of a broader range of environmental microorganisms, including a higher number of non-proteolytic strains. This result aligns with the known advantages of droplet-based cultivation, such as growth in liquid microenvironments and the absence of direct interspecies competition, which together improve access to cultivable microbes compared with conventional screening (15–17).

### Methodological details of IDO analysis of the sludge sample

**Fig 3:**
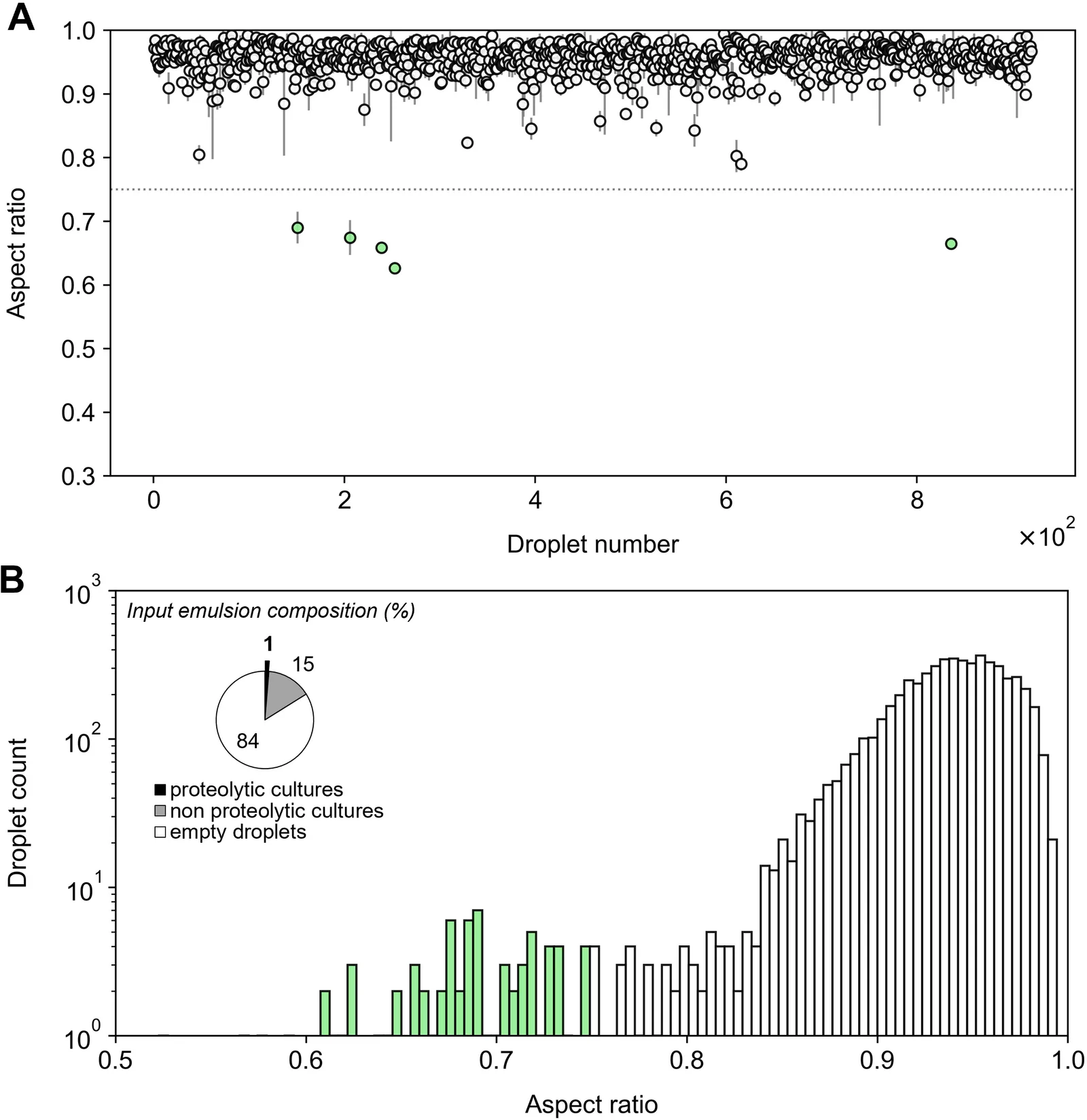
Detection of microbial proteolytic activity. (**A**) The figure summarizes the results of the image-based method applied to video recordings from enrichment experiments in the DPDS device. The scatter plot shows the mean aspect ratio of individual droplets. The error bars represent the standard deviation of the aspect ratio calculated across multiple frames for each droplet. Droplets with aspect ratio values below 0.75 (gray dotted line) were classified as liquid, indicating microbial proteolytic activity (green circles). (**B**) The bottom histograms display the aspect ratio distribution of droplets detected from the video recordings, with green bins representing droplets with an aspect ratio below 0.75, which were identified as positive droplets encapsulating individual proteolytic cultures. The emulsion composition (λ ~ 0.2) is illustrated in the pie chart, calculated from video recordings and IDO analysis of a total of 5,666 droplets.

Each droplet detected via IDO was calculated as the result of consecutive frames in which a full droplet appeared within a defined region of interest (ROI) in the video (21). During video analysis, all extracted data were saved in an output file (e.g., .csv or .xlsx) together with the corresponding screened frames, thereby documenting the detection of droplets within the ROI. In total, 5,666 droplets were automatically analyzed from video recordings to detect proteolytic colonies, Fig. 3A and B. IDO analysis was performed using strict area and perimeter thresholds to ensure that only droplets of approximately 100 pL were detected, thereby excluding debris, satellite droplets, and merged droplets from the data set, as shown in Fig. S1. To discriminate between negative solid and spherical droplets (empty or containing non-proteolytic cultures) and positive elongated droplets with proteolytic activity, we applied an aspect ratio threshold of 0.75. Droplets with an aspect ratio below this value were classified as positive. This threshold was determined in our previous study using LB 0.5× droplets with varying gelatin concentrations (0%–7.5%), as it corresponds to the solid-to-liquid transition observed between 3% and 4.5% gelatin (21). The sewage sludge emulsion composition (λ ~ 0.2) is shown in the pie chart. In addition to IDO analysis, proteolytic cultures were enumerated by visual inspection of video recordings to validate the classification. Empty droplets and non-proteolytic cultures, which were not automatically distinguished by IDO analysis, were visually identified and counted. In total, 5,666 droplets were classified.

### Enrichment of proteolytic consortia from sewage sludge

**Fig 4:**
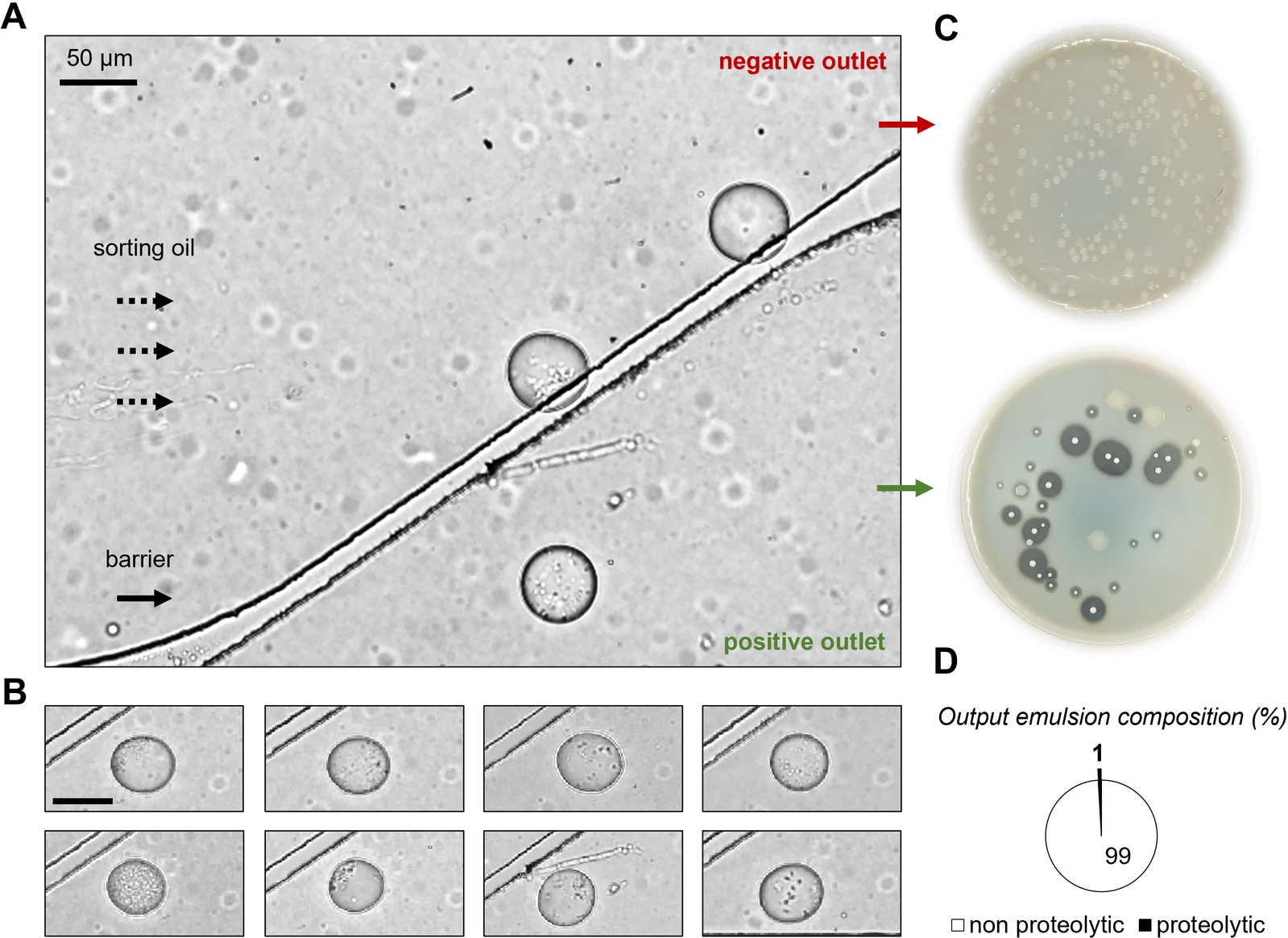
Isolation of proteolytic strains from sewage sludge. (**A**) Individual colonies were sorted simultaneously by the DPDS device: liquefied droplets were squeezed underneath the barrier by the laminar flow of oil and directed to the positive outlet. Empty droplets and those containing non-proteolytic strains, which remained solid and could not pass under the barrier, were directed to the negative outlet, scale bar: 50 µm. (**B**) Snapshots of positive droplets during sorting were captured during the enrichment, scale bar: 50 µm. (**C**) Droplets collected from both outlets were further characterized using SMA to assess sorter efficiency. Colonies recovered from positive droplets were predominantly proteolytic, except for a few possible non-proteolytic strains. In contrast, all colonies from negative droplets were non-proteolytic. (**D**) The output emulsion composition (%) was calculated based on the CFUs screened using the SMA protocol following DPDS enrichment.

Following the image-based analysis, we aimed to isolate proteolytic bacteria using the DPDS device, which sorts droplets in a passive manner and its performance was previously demonstrated for screening of mock microbial communities (22). Gelatin droplets with microbial colonies were sorted as shown in Fig. 4 A and B: liquefied droplets containing proteolytic strains were squeezed beneath the barrier and they were directed to the positive outlet, whereas empty droplets and those containing non-proteolytic strains remained solid and could not pass under the barrier. The screening was carried out at a controlled room temperature of 20°C. Droplet screening was performed for 240 min, which at a throughput of 50 droplets per second (50 Hz) corresponds to approximately 7.2 × 10⁵ sorted droplets. The DPDS outlets were connected via sterile Teflon tubing to sterile Eppendorf tubes in which the droplets were collected. Once the enrichment was completed, the tubes were disconnected from the device, and the emulsions were broken as described in Materials and Methods, resulting in the resuspension of bacteria in physiological solution. The bacterial suspension from merged droplets was divided, with a small fraction (50 µL) analyzed using the SMA test for both positive and negative outlet samples, and the remaining fraction (150 µL) processed for sequencing. SMA analysis of the DPDS outlets indicated a high level of sorter efficiency; however, cultivation on solid media is known to bias the representation of environmental microbial communities (16), this test should be regarded as a qualitative control (Fig. 4C). A few potential false positives were observed, as non-proteolytic colonies were found in the positive outlet sample. This estimate of approximately 10% false positives in Petri dishes should be interpreted with caution, as growth on solid agar can induce metabolic states that differ from those in liquid droplets. Moreover, the low variability in colony number and morphology observed on SMA among streaked droplet samples suggests that a substantial fraction of strains did not grow, rendering SMA screening poorly informative for accurate quantification at this post-droplet enrichment stage. In contrast, all colonies recovered from the negative outlet lacked proteolytic activity. The limited diversity in morphology and pigmentation of non-proteolytic colonies further indicates reduced variability, likely reflecting the dominance of fast-growing strains on solid media. The output emulsion composition (%), Fig. 4D, was assessed by streaking droplets sorted from the negative and positive outlets of the DPDS device onto SMA plates.

### Droplet-based cultivation of sewage sludge-derived microorganisms enhances microbial diversity and abundance compared with the traditional protocol

**Fig 5:**
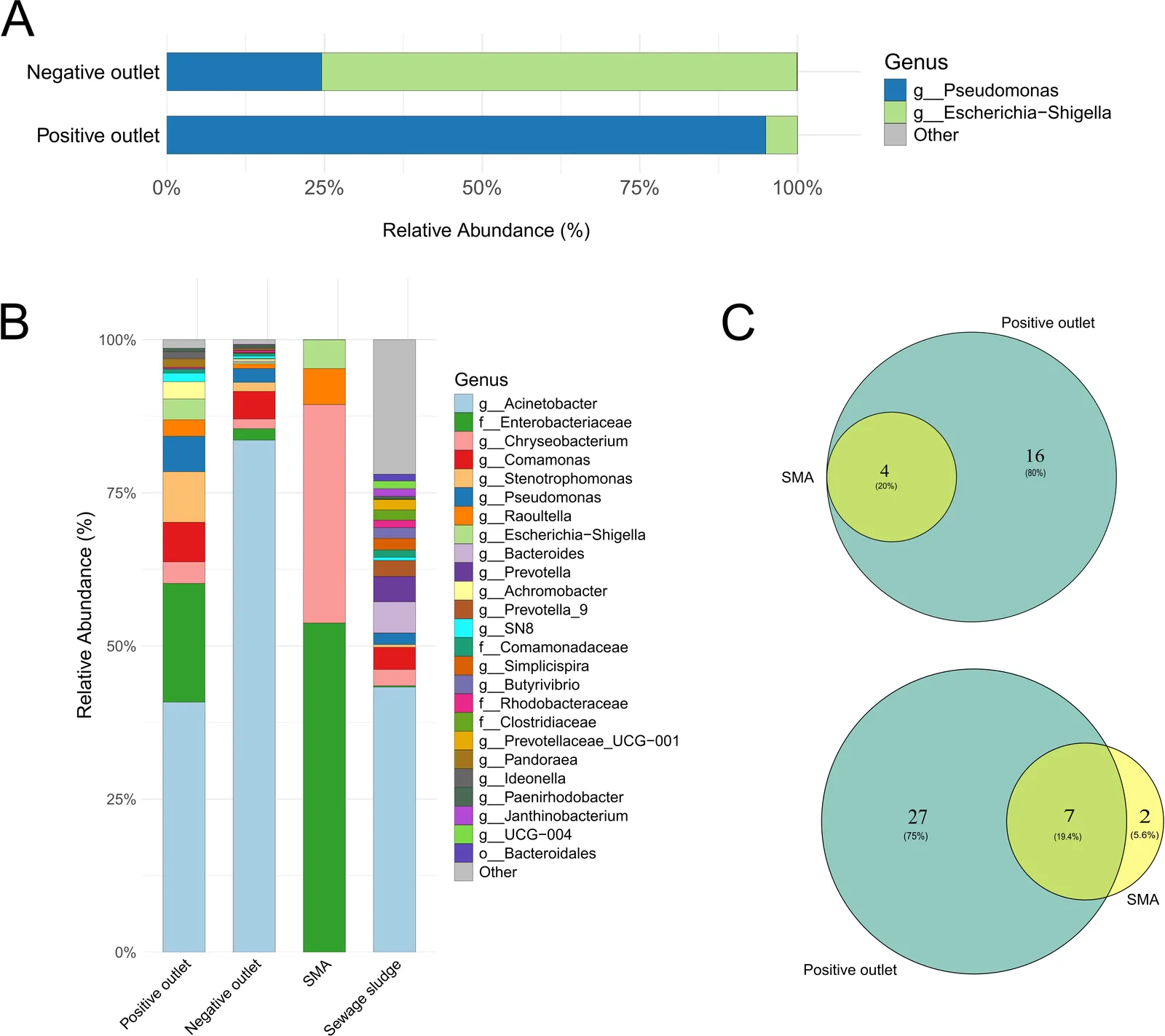
Comparison of microbial diversity recovered by microfluidic droplet-based enrichment and SMA plate culturing. (**A**) Genus-level taxonomic composition of the most abundant genera in the positive and negative droplet sorter outlets for mock consortia experiment. All remaining taxa are grouped as “Other.” (**B**) Genus-level taxonomic composition of the most abundant genera in original sludge, SMA plate isolates, and the positive and negative droplet sorter outlets. All remaining taxa are grouped as “Other.” (**C**) Venn diagrams showing the overlap of genera (up) and ASVs (down) recovered by SMA plating and the positive microfluidic outlet.

After validating the reliability of the device output, as described in the “Preliminary Test” section of the supplemental material (see Fig. 5A), we assessed its ability to recover proteolytic strains from a complex sewage sludge sample by comparing its performance with the conventional SMA cultivation method. The V4–V5 region of the 16S rRNA gene was sequenced (Illumina NovaSeq) from (i) droplets collected from both DPDS sorter outlets, (ii) proteolytic colonies isolated on SMA medium, and (iii) the original sewage sludge sample. The estimated number of droplets collected through the positive outlet of the DPDS was calculated based on the emulsion composition and the screening duration. During a 4-h screening, approximately 7.2 × 10⁵ droplets (100 pL each) were analysed. Of these, 1.3% were positive (~9,360 droplets), corresponding to a final collected volume of approximately 0.9 μL. In contrast, conventional screening on SMA medium yielded only 32 proteolytic colonies, hand-picked based on transparent halos.

To assess whether the microfluidic device recovered a higher diversity of proteolytic species than SMA plates, we compared the two samples using rarefaction–interpolation analysis (24) based on Hill numbers (*q* = 0, 1, 2) with 95% confidence intervals (25). Diversity extrapolation curves showed that the positive outlet exhibited substantially higher ASV richness than SMA cultivation, Fig. S2A, driven by a broader taxonomic composition enriched in rare and low-abundance taxa largely absent from plate-derived samples. Rank–abundance distributions confirmed higher evenness in droplet samples, whereas SMA plates were dominated by a few fast-growing taxa, Fig. S2B, consistent with the known advantages of physical compartmentalization in minimizing competitive interactions (16, 26, 27).

Sequencing of the sludge sample revealed 43.28% of reads assigned to *Acinetobacter*, followed by *Bacteroides* (5%); members of the *Prevotella* genus, such as *Prevotella* (4.1%) and *Prevotella-9*, *Comamonas* (3.6%), *Chryseobacterium* (2.6%), and several other genera, each representing <2% relative abundance as reported in Fig. 5B and Table S1. This pattern is consistent with previous studies reporting *Acinetobacter* as a frequent dominant genus in wastewater treatment plants, although its relative abundance varies depending on source, plant type, and operational conditions (28–33). SMA plates recovered only four genera: Enterobacteriaceae family (53.8%), *Chryseobacterium* (35.7%), *Raoultella* (5.1%), and *Escherichia–Shigella* (4.7%), with *Acinetobacter* (the dominant sludge genus) completely absent (Fig. 5B). All detected taxa belong to bacterial groups with documented proteolytic capabilities, including the production of extracellular and/or cell-associated proteases involved in the degradation of proteinaceous substrates (34–36). Droplet-based cultivation and sorting, in contrast, preserved sludge diversity more faithfully: both sorter outlets were dominated by *Acinetobacter* (40.8% positive, 83.6% negative), recovering 20–23 genera. In the positive outlet, following *Acinetobacter*, reads were assigned to the Enterobacteriaceae family (19.4%), *Stenotrophomonas* (8.2%), *Comamonas* (6.4%), *Pseudomonas* (5.8%), and *Chryseobacterium* (3.5%). Although a small fraction of genera may represent false positives, the majority are known proteolytic taxa (e.g., *Chryseobacterium*, *Pseudomonas*, *Acinetobacter*, *Staphylococcus*) (37–40). The occasional detection of non-proteolytic taxa, such as *Microvirgula* (0.2%), and members of Rhodobacteraceae (0.3%), aligns with observations from the mock community, where false positives accounted for only ~5% of reads (Fig. 5A). Crucially, all four genera isolated on SMA plates were found in the positive microfluidic output, with seven out of nine plate-derived ASVs successfully captured by the device, as shown in Fig. 5C.

Droplet-based enrichment enhanced low-abundance genera with relevant functional potential compared with conventional cultivation. In the sludge sample, 87.9% of detected taxa were below <1% relative abundance (Table S2). Ten low-abundance genera were recovered in droplets and/or plate cultures, six of which exceeded 1% abundance after enrichment (Table S3). Notably, all 10 were detected in the positive droplet outlet, whereas SMA screening recovered only two. Among the enriched genera, *Stenotrophomonas maltophilia* produces multiple secreted proteases (StmPR1/2/3) with strong proteolytic activity (41), while *Raoultella* and *Pandoraea* are associated with diverse protease systems and metabolic versatility relevant to protein degradation (42, 43). The enrichment of these taxa in droplets, particularly above 1% relative abundance, highlights the utility of droplet micro-compartmentalization for accessing taxa with *in situ* metabolic capacity that might otherwise be outcompeted in culture.

The negative outlet also yielded a substantial number of genera, with *Comamonas* as the second most abundant (4.5%), followed by *Pseudomonas* (2.2%), Enterobacteriaceae (1.8%), *Chryseobacterium* (1.6%), and *Stenotrophomonas* (1.6%); all other genera were present at <1% relative abundance (Fig. 5B and Table S1).

Overall, droplet-based microfluidic cultivation enabled a more comprehensive recovery of microbial diversity than conventional plate methods, substantially increasing the capture of proteolytic and functionally relevant species (including rare and slow-growing taxa) that are otherwise underrepresented in standard culture-based approaches.

### Conclusion

Droplet microfluidics enables ultra-high-throughput screening up to 10⁸ samples per day (44) with low reagent consumption and supports single-cell analyses, allowing detection of rare taxa and slow-growing strains (16). Nevertheless, the characterization of microbial proteolytic strains remains relatively underexplored within the context of microfluidic environmental screening (18). Our workflow provides a robust framework for the functional screening and taxonomic characterization of proteolytic microbes from sewage sludge, supporting a comprehensive understanding of their composition. In this study, we demonstrate that droplet-based microfluidic screening provides a powerful and scalable strategy for the functional analysis (IDO) and enrichment (DPDS) of proteolytic microbial consortia from environmental samples, such as sewage sludge. By combining single-cell encapsulation in gelatin microcompartments, label-free image-based detection of proteolytic activity, and passive droplet sorting of proteolytic cultures, we establish a comprehensive workflow that overcomes several inherent limitations of conventional bulk methods. This approach achieves substantially higher throughput and, more importantly, enables the screening of a larger number and greater diversity of microbial strains.

Importantly, when benchmarked against other droplet-based high throughput screening (HTS) workflows, our approach offers substantially greater practical simplicity. Existing droplet microfluidic platforms for environmental HTS typically rely on fluorescence-activated droplet sorting (FADS) to isolate proteolytic strains (45). Although these methods achieve higher throughput than our protocols, they require specialized substrates or fluorogenic reporters as well as complex optical systems (46). In contrast, the label-free, image-based detection strategy coupled with passive sorting eliminates the need for reporters and reduces system complexity, while maintaining throughput levels that, although lower than those of FADS, remain sufficiently high for large-scale microbial screening.

Fluorogenic substrate-based assays, including methods employing BODIPY-labeled substrates, currently represent some of the most established and sensitive strategies for droplet-based enzymatic screening. These approaches rely on synthetic fluorogenic substrates that generate a fluorescent signal upon enzymatic cleavage, enabling highly sensitive detection of proteolytic activity. However, the physicochemical properties of such synthetic substrates may differ substantially from those of natural proteinaceous materials and therefore may not fully reproduce native degradation processes. In contrast, the platform presented here directly monitors gelatin degradation through proteolysis-induced changes in droplet viscoelasticity. Additionally, the label-free nature of the method eliminates the need for substrate labeling or chemical modification, reducing the risk of altering substrate accessibility or enzymatic specificity.

We compared our workflow with SMA screening, a standard bulk method for detecting proteolysis. Solid-medium cultivation underestimated the abundance and diversity of proteolytic microorganisms, whereas droplet-based systems recovered a richer community, including taxa absent or underrepresented on plates, and captured a broader range of proteolytic genera.

The 16s rRNA sequencing of sorted droplet populations further demonstrated that microfluidic enrichment yields communities that more closely reflect the original sludge microbiome, while selectively enriching for proteolytic phenotypes. By incorporating sequencing directly after droplet sorting, we gained deeper insights into the ecological and biotechnological potential of these communities than approaches where sequencing follows traditional cultivation or bulk extraction, in which only a fraction of what grows in liquid droplets can be recovered on solid media (45). Future developments will focus on integrating the present high-throughput droplet screening platform with droplet deposition systems (47). Such coupling would bridge single-cell, in-droplet cultivation and selection with automated downstream upscaling in well plate formats (48).

A limitation of the present study is the lack of functional characterization of the recovered proteolytic isolates following droplet sorting and subsequent cultivation on SMA plates. Conventional solid-medium cultivation approaches may introduce substantial cultivation bias, which is prevented in droplet-based assays through single-cell compartmentalization. In highly complex environmental communities, such as sewage sludge, additional cultivation on Petri dishes may preferentially promote the growth of fast-growing and highly competitive microorganisms and induce substantial phenotypic variation, potentially altering the composition of the proteolytic communities initially enriched with droplets. Nonetheless, future works will focus on functional and molecular characterization of the droplet cultures recovered after sorting. Such studies will support the establishment of a collection of highly proteolytic strains with potential biotechnological relevance.

Additionally, this newly developed microfluidic method can be adapted to anaerobic conditions, as demonstrated by Watterson et al. (16), thereby opening potential applications for the screening of proteolytic consortia in anaerobic digestion systems aimed at biomethane production. Overall, this work further expands microfluidic methods, establishing them as a robust platform for functional HTS of microorganisms. The ability to selectively enrich active proteolytic microorganisms opens new opportunities for recovering strains with potential applications in biomass hydrolysis, bioremediation, and industrial enzyme discovery. Our approach provides a general framework for linking microbial function to community composition in complex ecosystems. Furthermore, our methods can be readily adapted to diverse screening campaigns, provided that the solid-to-liquid transition of droplets can be exploited to trigger deformability-based sorting, for example, to detect agarolytic activity.

## MATERIALS AND METHODS

### Resuspension of the environmental sample

Sewage sludge was sampled in August 2024 from a biogas facility in Wołomin, Poland, and stored at 4°C in a 5-L plastic container. Approximately 25 mL was transferred into 50-mL centrifuge tubes and centrifuged at 2,000 rpm for 2 min to sediment particulate matter and concentrate the sample. One milliliter of sediment was resuspended in 100-mL sterile physiological saline in a 500-mL Erlenmeyer flask and incubated at 25°C with shaking at 250 rpm for 12 h to facilitate sample resuspension. The suspension was subsequently passed through a 40-µm cell strainer mesh filter (VWR) to remove coarse particles, and the filtrate was collected for downstream experiments.

### Gelatin droplet generation, cultivation of microbes, and screening

Preliminary screening of sewage sludge on LB agar was used to estimate the CFU/mL of the sample. After overnight shaking in physiological solution, an appropriate volume of the cell suspension was transferred to a 1.5-mL microcentrifuge tube to achieve the desired cell-to-droplet ratio (λ) in the resulting emulsion. Previous relevant droplet-based microbiome studies have reported successful screening of clonal cultures at higher encapsulation loading concentrations, such as λ ~ 0.4 (18). In contrast, the lower loading concentration applied in the present study, λ ~ 0.15, further minimized co-encapsulation events and ensured that the majority of occupied droplets contained single microbial cells.

Cells were collected by centrifugation (5,000 × *g* for 5 min), the supernatant was discarded, and the cell pellet was thoroughly resuspended in gelatin medium. The medium was prepared in ddH₂O and consisted of 5 g/L NaCl, 2.5 g/L yeast extract, 5 g/L tryptone, and 75 g/L gelatin. The solution was then sterilized by autoclaving. Droplet emulsions were generated using a flow-focusing droplet generator with an oil phase containing 5% RAN 008-Fluorosurfactant at 25°C (RAN Biotechnologies). The emulsions were collected in a droplet chamber and incubated at 40°C for 96 h. A peristaltic pump supplied oxygen dissolved in the oil to the bacteria inside the droplets while preventing air bubbles from entering the chamber (21, 22). Detailed protocols for droplet generation and microbial cultivation are available in the supplemental material (Fig. S3). Methods for DPDS microfabrication, device setup, and IDO analysis are described in the referenced publications (21, 22).

### Characterization of proteolytic consortia in the input samples

Sludge was streaked on Petri dishes on the same day as droplet generation by inoculating 100 µL of different serial dilutions (10⁻², 10⁻³, 10⁻⁴) of the suspension, with six replicates per dilution, on SMA. The SMA medium was prepared and autoclaved with the following composition: yeast extract 1 g/L, tryptone 4 g/L, agar 15 g/L, and skimmed milk powder 30 g/L. Plates were incubated at 40°C, and positive colonies were manually picked. The sample was resuspended in a 1.5 mL Eppendorf tube containing 200 µL of physiological solution and stored at −20°C until sequencing.

### Image-based analysis of droplets encapsulating proteolytic cultures

After incubation, the emulsion was reinjected from the incubation chamber into the DPDS device for image-based analysis. IDO analysis was performed using an optimized Python script that processes high-speed video to extract shape-based features of droplets flowing through the flow focusing section of the device. The script applies background subtraction and contour detection, followed by morphometric analysis to quantify descriptors, such as droplet area, perimeter, and aspect ratio. Annotated video frames and quantitative measurements are saved, allowing downstream analysis of droplet deformation as a proxy for microbial proteolytic activity. Thresholds for IDO analysis were set as follows: droplets with an area between 0.6 × 10⁴ and 1.0 × 10⁴ pixels and a perimeter between 300 and 400 pixels were retained in the data set, excluding merged droplets, satellites, and debris significantly larger or smaller than the target 100pL droplets (Fig. S1).

### Enrichment of droplets and characterization of proteolytic consortia

Passive sorting of proteolytic microorganisms from the mock consortia experiments was performed using the DPDS device according to the protocols previously described (22), following the same experimental workflow subsequently applied for screening proteolytic microorganisms from sewage sludge. At the end of sorting, the tubing connected to the outlets was flushed with sterile, filtered HFE-7500 oil. The emulsions were broken by adding 20% (v/v) 1H,1H,2H,2H-perfluoro-1-octanol (PFO, Alfa Aesar) and 200 μL of sterile 0.9% NaCl solution. The biphasic mixtures were vortexed for 90 s and centrifuged for 60 s at 2,000 rpm to promote phase separation. The aqueous phases from the outlets were collected, stored for sequencing, and streaked in triplicate on SMA. Plates were incubated at 40°C and examined visually.

### DNA extraction, DNA amplification, and sequencing

DNA was extracted from six samples. These included (i) a sewage sludge sample, resuspended as described in the previous section; (ii) four droplet-derived samples; and (iii) one pooled sample consisting of 32 proteolytic colonies isolated using the SMA method. The four droplet samples were obtained after enrichment and sorting with the DPDS device, applied either to the environmental sludge or to a mock community composed of *Pseudomonas aeruginosa* (previously isolated from the same sludge) and *Escherichia coli* as a non-proteolytic control.

For each sample, DNA was extracted from 100 µL of starting material using the DNeasy Blood & Tissue Kit (Qiagen), following the manufacturer’s protocol for gram-negative bacterial cultures with an initial modification. Due to the low amount of cells in the droplet-derived samples, no pelleting step was performed to avoid material loss; instead, reagents were added directly to the liquid culture aliquots.

Extracted DNA was amplified using the universal prokaryotic primers 515F-Y (5′-GTGYCAGCMGCCGCGGTAA-3′) and 926R (5′-CCGYCAATTYMTTTRAGTTT-3′), targeting the V4–V5 hypervariable region of the 16S rRNA gene (49). PCRs were performed in triplicate using Phusion High-Fidelity DNA Polymerase (Thermo Fisher). Each 25-µL reaction contained 1 ng of template DNA, 1× Phusion HF buffer, 0.25 μM barcoded forward and reverse primers, 0.02 U/µL polymerase, and 200 μM of each dNTP. Cycling conditions consisted of an initial denaturation at 98°C for 4 min, followed by 20 cycles of 98°C for 20 s, 50°C for 30 s, and 72°C for 10 s, with a final extension at 72°C for 5 min. Triplicate reactions were pooled to minimize intra-sample variability and to obtain sufficient amplicon quantity, then purified using AMPure XP magnetic beads (Beckman Coulter).

Purified DNA was quantified using a NanoPhotometer NP80 (Implen), and fragment size distribution was assessed via 1% agarose gel electrophoresis. Samples were then sent to the University of Warsaw Sequencing Facility (CeNT Genomics Core Facility) for library preparation and sequencing on the NovaSeq 6000 next-generation sequencing platform (Illumina).

### Sequencing and analysis

Sequence quality was assessed using FastQC (50), and reads were processed in QIIME2. Primer sequences were removed with Cutadapt (51), and denoising was performed using the DADA2 pipeline to infer ASVs, including paired-end merging and chimera removal (Table S4) (52). Taxonomic assignment of the V4–V5 16S rRNA gene region was carried out using a Naive Bayes classifier trained on the SILVA 138.1 database and tailored to the 515F-Y/926R amplicon region (53, 54). Downstream analyses were conducted in R (version 4.2.0) using the phyloseq package (55) in RStudio 2023.09.0 (56). Rarefaction analyses conducted on unfiltered data indicated sufficient sequencing depth for all samples (Fig. S4A). A summary of raw ASV counts and read abundances prior to filtering is provided in Table S5. Prior to downstream analyses, ASVs classified as Eukaryota, chloroplasts, or mitochondria were removed, as were low-abundance ASVs to reduce the influence of spurious variants and improve the reliability of community structure assessments (Table S4). Rarefaction–extrapolation and rank–abundance analyses were then performed using the iNEXT package (24), and relative abundances were used for all comparative taxonomic analyses.

When exact taxonomic annotation was not achievable, sequences were assigned to the lowest reliably supported taxonomic rank for bacteria. Due to the intrinsic limitations of Illumina short-read sequencing, which provides insufficient phylogenetic resolution for deep-level discrimination, confident taxonomic assignment below the genus level was not feasible. Accordingly, strain-level classifications were avoided, as such assignments would be unreliable or inconsistent across samples.

## Data Availability

The research data used to obtain the results presented in the publication have been deposited in the University of Warsaw repository at the following address doi:10.58132/YZ3CEF. NGS data have been deposited in the EMBL-EBI European Nucleotide Archive (ENA) under the Project ID PRJEB107907.

## SUPPLEMENTAL MATERIAL

Supplemental material aem.00293-26-s0001.docx Supplemental methods, Fig. S1 to S4, and descriptive legends for supplemental tables and videos.

Supplemental tables aem.00293-26-s0002.xlsx Tables S1 to S5.

Video S1 aem.00293-26-s0003.mp4 IDO analysis of sewage sludge.

Video S2 aem.00293-26-s0004.mp4 DPDS enrichment of proteolytic cultures from sewage sludge.

ASM does not own the copyrights to Supplemental Material that may be linked to, or accessed through, an article. The authors have granted ASM a non-exclusive, world-wide license to publish the Supplemental Material files. Please contact the corresponding author directly for reuse.
