# Supplementary material for: Microfluidic enrichment of proteolytic microbial consortia from sewage sludge
Source: Appl Environ Microbiol. 2026 Jul 10;92(8):e00293-26. doi: 10.1128/aem.00293-26 (PMC13488334; doi:10.1128/aem.00293-26)
Supplement: Supplementary file 1 [file aem.00293-26-s0001.docx]

***Microfluidic enrichment of proteolytic microbial consortia from sewage sludge.***

Luca Potenza^a^, Valentina Smacchia^b^, Łukasz Drewniak^c^, Tomasz S. Kaminski^a^.

a - Laboratory of Microfluidics and Single Cell Analysis, Institute of Biochemistry, Faculty of Biology, University of Warsaw, Miecznikowa 1, 02-096 Warsaw, Poland

b - Institute of Evolutionary Biology, Faculty of Biology, University of Warsaw, Żwirki i Wigury 101, 02-089 Warsaw, Poland

c - Institute of Bioengineering, Faculty of Biology, University of Warsaw, Miecznikowa 1, 02-096 Warsaw, Poland.

**Table of Content**

**1. Supplemental methods**

- Droplet generation and microbial cultivation
- Sequencing and analysis

2. **Preliminary tests**

**3. Supplemental figures**

- Figure S1. Accuracy of IDO analysis
- Figure S2. Species richness across methods
- Figure S3. Schematic of the dynamic droplet incubation system
- Figure S4. Rarefaction analysis of microbial diversity

**4. Supplemental tables**

- Table S1. Distribution of ASVs and their relative abundances
- Table S2. Analysis of low-abundance (<1%) genera across samples.
- Table S3. Occurrence and enrichment of law-abundance genera from sludge samples across experimental conditions.
- Table S4. Summary of the bioinformatic processing and sequencing quality metrics.
- Table S5. Distribution of ASVs and their raw abundances before filtering.

**5. Supplemental videos**

- Video S1. IDO analysis of sewage sludge
- Video S2. DPDS enrichment of proteolytic cultures from sewage sludge

**1. Supplemental methods**

**Droplet generation and microbial cultivation.** Droplets were generated in a single-layer flow-focusing device (50 µm width × 50 µm height junction). The continuous phase (HFE oil containing 5% RAN 008-Fluorosurfactant) and the dispersed phase (0.5× LB supplemented with 7.5% gelatine and cells) were injected using Cetoni syringe pumps at 15 and 5 µL/min, respectively, yielding 100 pL droplets at a throughput of 1.7 kHz. Droplet incubation chambers were fabricated following Neun et al.[[1]](https://www.zotero.org/google-docs/?H6BlyV) using a 0.5 mL Eppendorf tube perforated with a 1 mm biopsy punch (Kai Medical). The tube lid was glued to a 1 mm thick glass slide, and two 20 cm PTFE tubes (0.4 mm I.D., 0.9 mm O.D., Bola Bohlender) were inserted and sealed. During droplet generation, the flow-focusing chip was connected to the upper tubing, allowing buoyant droplets to accumulate in the upper chamber. Emulsions were incubated for 96 hours using dynamic droplet incubation (DDI)[[2]](https://www.zotero.org/google-docs/?thXHNh). Oxygenation was provided by a peristaltic pump (Reglo ICC, Ismatec) connected via Tygon and PTFE tubing and coupled to a bubble trap filled with 5% fluorosurfactant in HFE-7500 to prevent air entry. The pump flow rate was set to 10 µL/min[[3]](https://www.zotero.org/google-docs/?wwbWW5). The experimental setup is shown in Figure S2.

**Sequencing and analysis.** Sequence quality was assessed using FastQC[[4]](https://www.zotero.org/google-docs/?cPxmz3). Reads were then imported into the QIIME2 environment, where sequencing primers were removed with Cutadapt[[5,6]](https://www.zotero.org/google-docs/?sT9okh). Denoising was performed with the DADA2 pipeline using the dada2 denoise-paired function in QIIME2, with a minimum overlap of 10 bp, no allowed mismatches, and chimera removal set to the consensus method[[7]](https://www.zotero.org/google-docs/?GZggms). Each sample was processed independently, and after denoising, amplicon sequence variant (ASV) tables and feature tables were merged. A total of 764796 reads were obtained after Cutadapt, with an average of 77126 to 352613 reads per sample; 81.5% of the reads completed the merging and chimera elimination filtering procedures (Table S1).

Taxonomic assignment of the V4–V5 16S rRNA gene region was carried out using a Naive Bayes classifier pre-trained on the SILVA 138.1 database[[8,9]](https://www.zotero.org/google-docs/?Tovnty). Reference sequences were trimmed to retain only the region spanning 200–600 bp, corresponding to the 515F-Y/926R amplicon, ensuring that the classifier was specifically tailored to the target region. The classifier was generated with RESCRIPt following guidelines provided on the Qiime forum page[[10,11]](https://www.zotero.org/google-docs/?7k5jiG). All taxonomic and diversity analyses were conducted in R (version 4.2.0) using RStudio 2023.09.0[[12]](https://www.zotero.org/google-docs/?RTDv2e). ASV tables and associated metadata were imported and managed using the *phyloseq* package[[13]](https://www.zotero.org/google-docs/?yTh2Aw), with data conversion from QIIME2 outputs performed via *qiime2R*[[14]](https://www.zotero.org/google-docs/?0zxBuh). Rarefaction curves were generated using the *vegan* package[[15]](https://www.zotero.org/google-docs/?LcMLpE) for all samples on the unfiltered dataset, indicating that sequencing depth was adequate (Figure S2). From the 16S dataset, a total of 1430 ASVs were initially inferred. Before downstream analyses, ASVs classified as Eukaryota, chloroplasts, or mitochondria were removed, yielding a dataset of 1425 ASVs. ASVs represented by fewer than 50 reads per sample were then removed to reduce the influence of spurious variants generated by sequencing noise or DADA2 over-splitting, yielding a final dataset of 372 ASVs (Table S1). Rarefaction–extrapolation and rank–abundance analyses were performed on this filtered ASV table using the *iNEXT* package[[16]](https://www.zotero.org/google-docs/?n3IGdK), and general taxonomic analyses were performed on non-normalized ASV counts transformed into relative abundances. Prokaryotic prevalence and community structure analyses were conducted using the *microbiome*[[17]](https://www.zotero.org/google-docs/?YeR4i9) and *microViz*[[18]](https://www.zotero.org/google-docs/?9uHwPQ) packages. Venn diagrams illustrating shared and unique ASVs among sample groups were generated using the *VennDiagram* package[[19]](https://www.zotero.org/google-docs/?8jlFnG). Data visualization was carried out using *ggplot2*[[20]](https://www.zotero.org/google-docs/?fPBnli), with additional graphical layout refinements implemented using the *grid* package[[21]](https://www.zotero.org/google-docs/?nxTKXS) for Venn diagrams illustrations.

When exact taxonomic annotation was not achievable, sequences were assigned to the lowest reliably supported taxonomic rank for bacteria. Due to the intrinsic limitations of Illumina short-read sequencing, which provides insufficient phylogenetic resolution for deep-level discrimination, confident taxonomic assignment below the genus level was not feasible. Accordingly, strain-level classifications were avoided, as such assignments would be unreliable or inconsistent across samples.

**2. Preliminary tests**

**Validation of sorting of proteolytic cultures from a binary mock community.** To assess the performance of the microfluidic enrichment performed with DPDS device, we sequenced the V4-V5 region of the 16S rRNA gene on an Illumina NovaSeq platform from a binary mock community composed of *Pseudomonas aeruginosa* (λ~0.01), a highly proteolytic reference strain previously isolated from the sewage sludge, and *Escherichia coli* (λ~0.1) as the non-proteolytic counterpart, with λ represents the mean cell occupancy per droplet under Poisson loading conditions. Based on the emulsion composition and the screening duration, the estimated total number of droplets collected from the DPDS outlets was approximately 3.6 × 10⁵ droplets (100 pL each) during a 2-hour screening (7.2 × 10³ s at 50 Hz). The sequencing analysis revealed that the DPDS device effectively enriched the proteolytic *Pseudomonas aeruginosa*. In fact, approximately 95% of reads assigned to *Pseudomonas* and *Escherichia-Shigella* were detected at minimal relative abundance (5%), indicating a very low rate of false positives. Conversely, the negative outlet showed 4 ASVs distributed across three genera, with a predominance of *Escherichia-Shigella* (75%) and approximately a 25% relative abundance of false negatives represented by *Pseudomonas,* Figure 5 (A). The relatively high rate of false negatives may be attributed to phenotypic variation. In previous experiments, a gradual loss of proteolytic activity in *P. aeruginosa* was observed during cultivation in gelatine droplets, suggesting instability of the expressed phenotype over time. Similar effects are common in conventional screening approaches, where it is recommended to revive cultures from glycerol stocks after several passages to minimise phenotypic drift. This effect may be further amplified in droplet-based assays, given the high-throughput nature of the process and the large number of screened events. The *P. aeruginosa* strain used in this study was isolated from sludge by selecting a single positive colony on SMA[[22-24]](https://www.zotero.org/google-docs/?66coJL). As an environmental isolate, it is likely subject to greater phenotypic variability than a stabilised *E. coli* line, leading to subpopulations that lose proteolytic activity. Consequently, some *P. aeruginosa* droplets remain solid, preventing their correct sorting under the optimized conditions. By contrast, the laboratory strain we tested *E. coli* DH5α exhibits markedly higher genetic and phenotypic stability. Such stability renders it considerably less prone to phenotypic variation than wild-type or environmental isolates, which often display heterogeneous behaviour under stress or during prolonged cultivation. Overall, these results confirm that sorting based on the positive outlet reliably enriches proteolytic strains, demonstrating the effectiveness of the DPDS device in selecting the desired phenotypes despite some unavoidable phenotypic variability.

**3. Supplemental figures**

**Figure S1. Accuracy of IDO analysis.** Histograms illustrating the performance of the IDO analysis applied to video recordings acquired during enrichment experiments in the DPDS device. The distributions demonstrate the accuracy of individual droplet detection. Discrimination thresholds based on droplet area (**A**) and perimeter (**B**) were implemented to exclude satellite and merged droplets from the analysis.

**Figure S2. Species richness across methods.** **(A)** Rarefaction–extrapolation curves based on Hill numbers (q = 0, q = 1, and q = 2) showing species richness for the positive microfluidic outlet and the SMA plate. Shaded areas indicate 95% confidence intervals, continuous lines indicate interpolated data, and dotted lines indicate extrapolated ones. (**B**) Rank–abundance plots highlighting differences in community evenness between the two methods. Relative abundances on the axis y are shown in a logarithmic scale.

**Figure S3. Schematic of the dynamic droplet incubation system.** The oxygenation system consisted of a closed-loop configuration designed to maintain a controlled oil flow. (**A**) A 0.5 mL Eppendorf tube serving as the droplet incubation chamber was affixed to a glass slide. (**B**) One tubing was connected to the upper part of the chamber, (**C**) while a second tubing was inserted near the lid. Both lines were sealed using PTFE tubing and connected to a peristaltic pump (Reglo ICC, Ismatec). The outlet tubing from the pump was connected to the lid of a second (**D**) Eppendorf tube containing 5% fluorosurfactant in filtered Novec HFE-7500, forming a closed loop. Oil was circulated at 10 µL/min, flowing from the top of the chamber, where droplets accumulated, towards the lower inlet.

**Figure S4. Rarefaction analysis of microbial diversity.** The rarefaction curves before filtering out non-bacterial taxa and low-abundance ASVs (**A**) for all samples, and (**B**) Focus on the rarefaction curves for all samples, excluding the environmental one. DMC_N and DMC_P represent negative and positive outlet samples for the mock community. ENV_W, DW_P, DW_N, and P_W represent sewage sludge, positive and negative outlet from sewage sludge, and SMA samples, respectively.

**4. Supplemental tables**

Supplemental tables are available in “supplemental_tables.xlsx”. The file contains the following tables:

**Table S1.** **Distribution of ASVs and their relative abundances.** Data includes the complete taxonomic lineage and the proportional representation of each variant identified in the experimental groups after taxa and low-abundance filtering.

**Table S2.** **Analysis of low-abundance (<1%) genera across samples.** The table reports the number of genera falling below the filtering threshold, the total number of genera identified per sample, and the corresponding percentage of low-frequency variants within each microbial community.

**Table S3.** **Occurrence and enrichment of low-abundance genera from sludge samples across experimental conditions.** The table lists genera with low initial abundance (<1%) in the sludge source that were also detected in droplets or petri dish samples. Columns specify the samples where these taxa were identified (Present_in) and those where they reached an enriched abundance of >1% (above 1%). Sample identifiers are as follows: DW_P (sludge-positive droplets), DW_N (sludge-negative droplets), and P_W (SMA).

**Table S4. Summary of the bioinformatic processing and sequencing quality metrics.** The table displays read counts and ASV yields across key pipeline stages including filtering, denoising, merging, and chimera removal for mock consortia, sludge samples, and SMA.

**Table S5.** **Distribution of ASVs and their raw abundances.** Data includes the complete taxonomic lineage and the representation of each variant identified in the experimental groups before filters.

**5. Supplemental videos**

**Video S1. IDO analysis of sewage sludge.** The video shows a fragment of one of the recordings used for image-based analysis of an emulsion containing 100 pL droplets of sewage sludge microcultures originating from single-cell encapsulation (λ~0.2). The oil flow is directed from left to right. The recording shows different types of droplets: empty solid droplets, solid droplets containing non-proteolytic cultures, and liquid droplets containing proteolytic cultures, subjected to the squeezing oil while flowing through the flow-focusing junction. The video was recorded at 7500 frames per second with a shutter speed of 1/80000 s and was slowed down by approximately 140 times.

**Video S2. DPDS enrichment of proteolytic cultures from sewage sludge.**The video shows the enrichment of a proteolytic culture from an emulsion containing 100 pL droplets of sewage sludge microcultures originating from single-cell encapsulation (λ~0.2). The oil flow is directed from left to right. The recording shows different types of droplets: solid droplets that are either empty or contain non-proteolytic cultures, and a liquid droplet containing a proteolytic culture. Droplets are sorted according to their deformability as they flow against the DPDS microbarrier. The video was recorded at 5000 frames per second with a shutter speed of 1/50000 s and was slowed down by approximately 160 times.

***References***

[1] Neun S, Kaminski TS, Hollfelder F. Chapter Five - Single-cell activity screening in microfluidic droplets. In: Allbritton NL, Kovarik ML, editors. Enzyme Act. Single Cells, vol. 628, Academic Press; 2019, p. 95–112. https://doi.org/10.1016/bs.mie.2019.07.009.

[2] Mahler L, Tovar M, Weber T, Brandes S, Rudolph MM, Ehgartner J, et al. Enhanced and homogeneous oxygen availability during incubation of microfluidic droplets. RSC Adv 2015;5:101871–8. https://doi.org/10.1039/C5RA20118G.

[3] Potenza L, Andrzejewski MS, Kaminski TS. Induced droplet ovalization (IDO): Image-based microfluidic method for high-throughput and label-free characterization of microbial proteolytic strains from wastewater sludge. Sens Actuators B Chem 2025;445:138554. https://doi.org/10.1016/j.snb.2025.138554.

[4] Andrews S. A Quality Control Tool for High Throughput Sequence Data, 2010. Available online at. https://www.bioinformatics.babraham.ac.uk/projects/fastqc/.

[5] Martin M. Cutadapt removes adapter sequences from high throughput sequencing reads. EMBnet J 2011:17:10. https://doi.or g/10.14806/ej.17.1.200.

[6] Bolyen E, Rideout JR, Dillon MR et al. Reproducible, interactive, scalable and extensible microbiome data science using QIIME 2. Nat Biotechnol 2019;37:852–7. https://doi.org/10.1038/s41587-019-0209-9.

[7] Callahan BJ, McMurdie PJ, Rosen MJ, Han AW, Johnson AJA, Holmes SP. DADA2: High-resolution sample inference from Illumina amplicon data. Nat Methods 2016;13:581–3. https://doi.org/10.1038/nmeth.3869.

[8] Pruesse E, Quast C, Knittel K, Fuchs BM, Ludwig W, Peplies J, et al. SILVA: a comprehensive online resource for quality checked and aligned ribosomal RNA sequence data compatible with ARB. Nucleic Acids Res 2007;35:7188–96. https://doi.org/10.1093/nar/gkm864.

[9] Quast C, Pruesse E, Yilmaz P, Gerken J, Schweer T, Yarza P, et al. The SILVA ribosomal RNA gene database project: improved data processing and web-based tools. Nucleic Acids Research 2012;41:D590–6. https://doi.org/10.1093/nar/gks1219.

[10] Robeson MS II, O'Rourke DR, Kaehler BD, Ziemski M, Dillon MR, Foster JT, et al. RESCRIPt: reproducible sequence taxonomy reference database management. PLoS Computational Biology 2021;17:e1009581. https://doi.org/10.1371/journal.pcbi.1009581.

[11] Robeson MS, O’Rourke DR, Kaehler BD, Ziemski M, Dillon MR, Foster JT, et al. RESCRIPt: Reproducible sequence taxonomy reference database management. PLoS Comput Biol 2021;17:e1009581. https://doi.org/10.1371/journal.pcbi.1009581.

[12] Allaire J. RStudio: integrated development environment for R. Boston: The R Project for Statistical Computing 2012;Vol. 770:165–71.

[13] McMurdie PJ, Holmes S. phyloseq: An R Package for Reproducible Interactive Analysis and Graphics of Microbiome Census Data. PLoS ONE 2013;8:e61217. https://doi.org/10.1371/journal.pone.0061217.

[14] QIIME 2 Forum. Tutorial: integrating QIIME 2 and R for data visualization and analysis using qiime2R. QIIME 2 Forum 2018. https://forum.qiime2.org/t/tutorial-integrating-qiime2-and-r-for-data-visualization-and-analysis-using-qiime2r/4121

[15] Oksanen J, Simpson G, Blanchet F, al et. Vegan: community ecology package. CRAN 2023. https://github.com/vegandevs/vegan.

[16] Hsieh TC, Ma Kh, Chao A. iNEXT: an R package for rarefaction and extrapolation of species diversity (H ill numbers). Methods Ecol Evol 2016;7:1451–6. https://doi.org/10.1111/2041-210X.12613.

[17] Lahti L, Shetty S. Microbiome R package. Boston Bioconductor 2017. https://doi.org/10.18129/B9.bioc.microbiome.

[18] Barnett, al et. microViz: an R package for microbiome data visualization and statistics. Journal of Open Source Software, 6(63), 3201, https://doi.org/10.21105/joss.03201. J Open Source Softw 2021;6(63):3201. https://doi.org/10.21105/joss.03201.

[19] Chen H, Boutros PC. VennDiagram: a package for the generation of highly-customizable Venn and Euler diagrams in R. BMC Bioinformatics 2011;12:35. https://doi.org/10.1186/1471-2105-12-35.

[20] Wickham H. ggplot2: Elegant Graphics for Data Analysis. N Y Springer Int Publ 2016. https://ggplot2.tidyvers e.org.

[21] Murrell P. R graphics. Chapman and Hall/CRC; 2005.

[22] Scheffler RJ, Bratton BP, Gitai Z. Pseudomonas aeruginosa clinical blood isolates display significant phenotypic variability. PLoS One 2022;17:e0270576. https://doi.org/10.1371/journal.pone.0270576.

[23] Grosso-Becerra M-V, Santos-Medellín C, González-Valdez A, Méndez J-L, Delgado G, Morales-Espinosa R, et al. Pseudomonas aeruginosa clinical and environmental isolates constitute a single population with high phenotypic diversity. BMC Genomics 2014;15:318. https://doi.org/10.1186/1471-2164-15-318

[24] Workentine ML, Sibley CD, Glezerson B, Purighalla S, Norgaard-Gron JC, Parkins MD, et al. Phenotypic heterogeneity of Pseudomonas aeruginosa populations in a cystic fibrosis patient. PloS One 2013;8:e60225. https://doi.org/10.1371/journal.pone.0060225.
